# Catheter ablation for persistent atrial fibrillation in an elderly patient with cor triatriatum sinister

**DOI:** 10.1016/j.hrcr.2022.06.009

**Published:** 2022-07-03

**Authors:** Shuko Iwata, Masaru Yamaki, Keita Nakagawa, Shuntaro Higuchi, Hirotsuka Sakai, Yuichiro Kawamura

**Affiliations:** ∗ Department of Cardiovascular Medicine, Nayoro City General Hospital, Nayoro, Japan; † Department of Cardiovascular Medicine, National Hospital Organization Obihiro Hospital, Obihiro, Japan; ‡ Health Administration Center, Asahikawa Medical University, Asahikawa, Japan

**Keywords:** Cor triatriatum sinister, Atrial fibrillation, Catheter ablation, Elderly patient, Pulmonary vein isolation, Congenital heart disease, Multidetector computed tomography

**Key Teaching Points**

• Three-dimensional images must be evaluated before and during catheter ablation among patients with cor triatriatum sinister.
• Pulmonary vein isolation is important, and additional ablation may be essential if extra–pulmonary vein trigger-related factors or tachycardia is induced.
• Catheter ablation for atrial fibrillation is significantly effective in relieving symptoms in patients in their 80s.

## Introduction

Cor triatriatum sinister (CTS) accounts for <0.1% of all congenital heart diseases. It is a condition in which the fibromuscular membrane divides the left atrium (LA) into 2 chambers.^1^ The superior and posterior chambers receive the pulmonary veins, and the inferior and anterior chambers are connected to the left atrial appendage and mitral orifice.^2^ Pathophysiologically, CTS is similar to mitral stenosis,^3^ and the symptoms are correlated with pulmonary venous congestion and pressure loading at the right side of the heart.^1^ Atrial fibrillation (AF) is caused by atrial overload, and AF occurs in 32% of adult patients with CTS.^4^ Since 2009, 14 cases of catheter ablation (CA) for AF in patients with CTS have been described, and the mean age of the patients was 64 ± 8 (range: 51–77) years.^5^ However, CA in patients aged over 80 years with CTS has never been reported, to the best of our knowledge. The current case report aimed to present the efficacy of CA for persistent AF in an elderly patient with CTS.

## Case report

**Figure 1:**
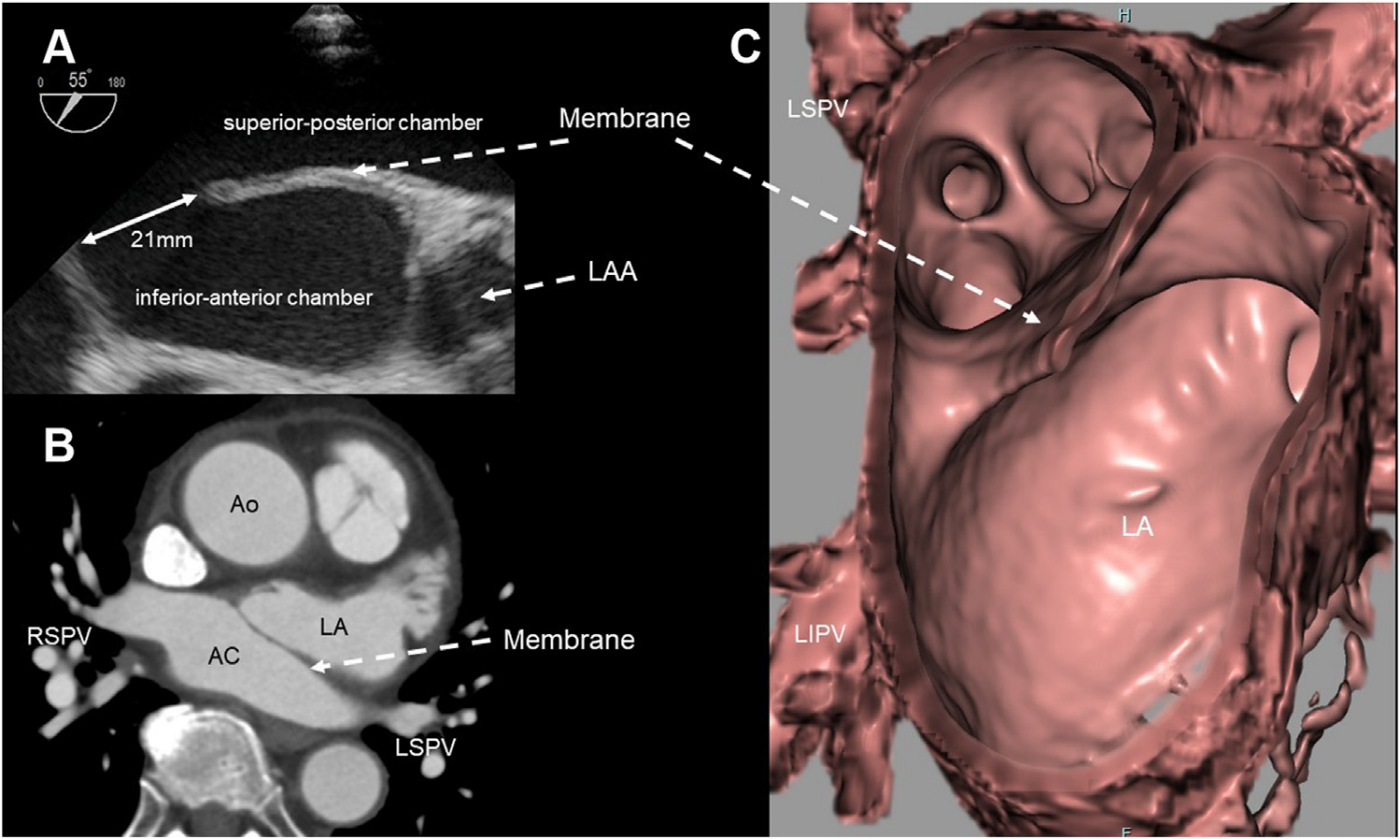
Images obtained via transesophageal echocardiography (**A**) and multidetector computed tomography (**B, C**). The fibromuscular membrane divided the left atrium into 2 chambers. The pulmonary veins opened into the superior and posterior accessory chambers, connected via the fibromuscular membrane to the inferior and anterior chamber and the main left atrial chamber. AC = accessory chamber; Ao = aorta; LA = left atrium; LAA = left atrial appendage; LIPV = left inferior pulmonary vein; LSPV = left superior pulmonary vein; RSPV = right superior pulmonary vein.

An 81-year-old Japanese male patient presented with persistent AF for more than 2 months. His symptoms were palpitations, pitting edema, fatigue, and dyspnea. He was then treated with loop diuretics, beta blockers, and digitalis at a local hospital. A transthoracic echocardiogram showed a left ventricular ejection fraction of approximately 50%, mild aortic valve stenosis, pericardial effusion, and the fibromuscular membrane in the LA. The diameter of the LA was 48 mm. Multidetector computed tomography (MDCT) and transesophageal echocardiography revealed a small atrial septal defect on the anterior side and a 3-mm-thick membrane dividing the LA into 2 chambers (Figure 1). The flow velocity between the 2 chambers was 84.1 cm/s, and the pressure gradient was 2.8 mm Hg. The patient’s symptoms persisted despite treatment. Hence, he underwent CA for AF. All procedures complied with the ethical standards of the responsible committee on human experimentation and with the 1975 Declaration of Helsinki, revised in 2000. The patient provided written informed consent.

**Figure 2:**
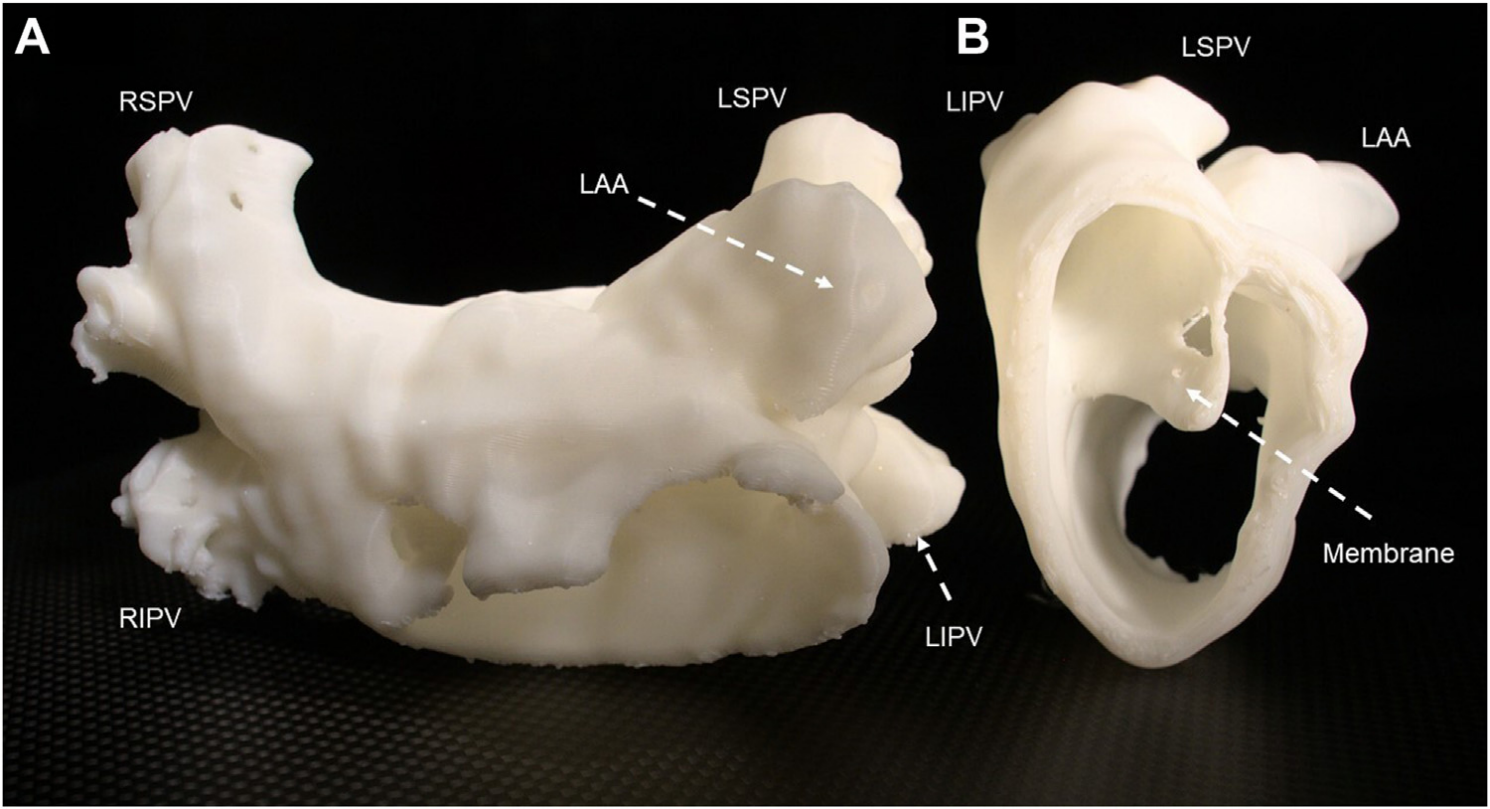
A model of the left atrium using the multidetector computed tomography data created via a 3D printer. **A:** Anterior-posterior view. **B:** Sagittal view. LAA = left atrial appendage; LIPV = left inferior pulmonary vein; LSPV = left superior pulmonary vein; RIPV = right inferior pulmonary vein; RSPV = right superior pulmonary vein.

**Figure 3:**
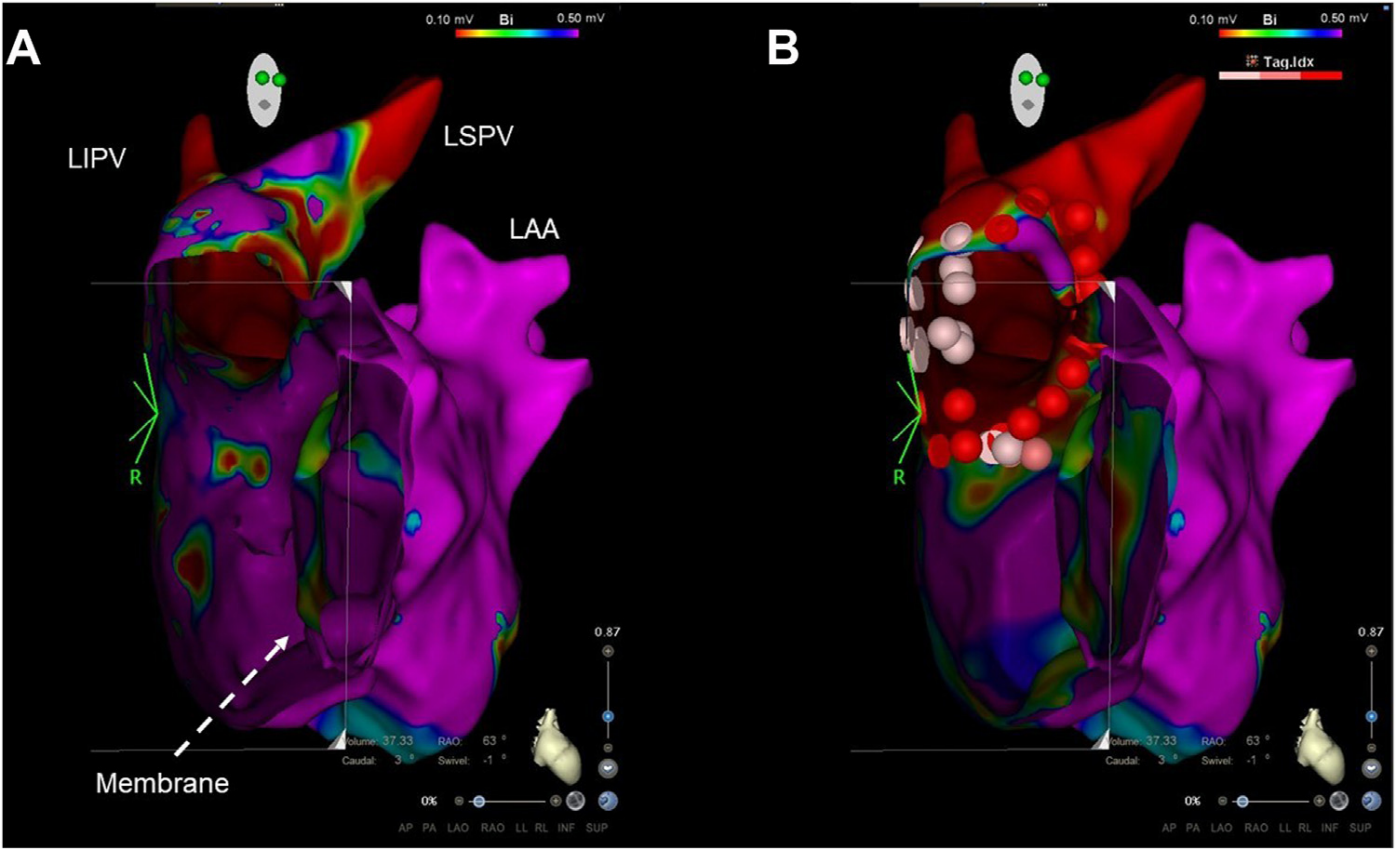
Voltage map (right anterior oblique view) of the left pulmonary vein before (**A**) and after (**B**) ablation. LAA = left atrial appendage; LIPV = left inferior pulmonary vein; LSPV = left superior pulmonary vein.

At preprocedure, we made a model of the LA using the MDCT data via the 3D printer. Furthermore, we made a reference to the Brockenbrough point and the ablation line (Figure 2A and 2B). The patient underwent a double transseptal puncture in the posterior side to access the LA. After a transseptal puncture, a sinus rhythm LA voltage map was created with a 3D electroanatomic mapping system (CARTO; Biosense Webster, Irvine, CA) via intracardiac echocardiography (SOUNDSTAR; Biosense Webster) and with a high-density multipolar mapping catheter (PENTARAY; Biosense Webster) (Figure 3A). A 3.5-mm-tip ablation catheter (ThermoCool; Biosense Webster) was used in extensive encircling pulmonary vein isolation (EEPVI). The endpoint of pulmonary vein (PV) isolation was a bidirectional conduction block between the PV and LA. To evaluate the ablation line, voltage mapping was performed with a multipolar mapping catheter (Figure 3B). After EEPVI, superior vena cava isolation was conducted. At the end of the procedure, isoproterenol was administered to analyze the extra-PV trigger-related factor. However, this was not documented. Rapid atrial pacing did not induce any tachycardia. The patient was discharged 3 days after the procedure with no complications. During the 18-month follow-up, he presented with persistent sinus rhythm without treatment with any antiarrhythmic agents, and all symptoms improved.

## Discussion

To the best of our knowledge, this report first described the use of CA in a patient who was in his 80s and presented with persistent AF. The symptoms correlated with congestive heart failure (CHF) persisted despite treatment with medications. A previous study showed that rhythm control therapy has clinical benefits for patients within 1 year after AF diagnosis.^6^ Hence, in this case, CA was performed. Then, PV isolation and superior vena cava isolation were conducted using the 3D electroanatomic mapping system. During the 18-month follow-up, the patient presented with sinus rhythm without any treatment with antiarrhythmic agents, and all symptoms improved.

Since Yamada and colleagues^7^ first reported successful CA for AF in a patient with CTS in 2008, 14 cases were published until 2021. All patients underwent PV isolation and became arrhythmia-free, with a success rate of >90%.^5^ However, it remains unknown whether procedures other than PV isolation, such as mitral isthmus blocking and roofline and anterior line ablation, were performed. Borne and colleagues^8^ conducted additional roofline ablation and mitral isthmus ablation because roof-dependent atrial flutter and perimitral flutter were induced by rapid atrial pacing after PV isolation. Okada and colleagues^9^ reported a case of focal atrial tachycardia with reentrant circuit appearing as a figure of 8 and in the anterior chamber. Karimianpour and colleagues^5^ found a low-voltage zone inside the fibromuscular membrane and substrate for organized atrial tachycardia. This finding indicated that the fibromuscular membrane was correlated with AF reentry, making ablation line beyond the PV isolation necessary. In this case, AF lasted for several months, and extra-PV trigger-related factors and tachycardia were not induced after EEPVI. Hence, ablation other than EEPVI was not performed.

Owing to severe membrane obstruction, CHF, and thriving difficulties, CTS is diagnosed in infancy in most cases.^10^, ^11^, ^12^ At the time of CTS diagnosis in adulthood, 82.5% of patients are symptomatic. In patients with CTS, their symptoms are AF (32.8%), CHF-related symptoms (26.9%), thromboembolic events (15.8%), and pulmonary hypertension (14.6%). The median age at CTS diagnosis in adults without membrane obstruction is 50 (range: 32–64) years.^4^ However, in this case, the patient did not present with symptoms for more than 80 years until the development of AF. Modi and colleagues^13^ showed that patients with an orifice diameter of >1 cm are symptom-free until adulthood. In this case, the fact that the patient has a 21-mm-diameter orifice (half the size of the LA dimension) and an atrial-septal defect might be one of the reasons that the pressure gradient was not very high and the patient was symptom-free until his 80s.

CTS is associated with different anatomical abnormalities.^14^ Hence, CA is challenging to perform on patients with CTS. Fukumoto and colleagues^15^ first showed successful CA via 3D MDCT imaging merged with the electroanatomical map on CARTO. Hence, the anatomical structure must be assessed via 3D imaging before and during CA. The CARTO system could provide valuable information about complex congenital heart disease even in the current case. Thus, ablation could be safely performed. To successfully conduct CA in patients with PTS, MDCT and transthoracic and transesophageal echocardiography must be performed before the procedure. Moreover, a detailed LA map should be created with a 3D electroanatomic mapping system via intracardiac echocardiography and a high-density multipolar mapping catheter.

## Conclusion

CA can be safely performed even in patients with CTS aged >80 years if MDCT and transthoracic and transesophageal echocardiography are performed at preprocedure, and if a detailed LA map is created with a 3D electroanatomic mapping system via intracardiac echocardiography and a high-density multipolar mapping catheter. Moreover, CA effectively relieves symptoms among elderly patients with PTS who presented with persistent AF.
